# Computer hardware for radiologists: Part 2

**DOI:** 10.4103/0971-3026.73527

**Published:** 2010-11

**Authors:** IK Indrajit, A Alam

**Affiliations:** Department of Radiodiagnosis and Imaging, Command Hospital (Air Force), Bangalore - 560 007, India

**Keywords:** Bus, Computers, CT, drive interface, hardware, MRI, input/output device, port, storage drive

## Abstract

Computers are an integral part of modern radiology equipment. In the first half of this two-part article, we dwelt upon some fundamental concepts regarding computer hardware, covering components like motherboard, central processing unit (CPU), chipset, random access memory (RAM), and memory modules. In this article, we describe the remaining computer hardware components that are of relevance to radiology. “Storage drive” is a term describing a “memory” hardware used to store data for later retrieval. Commonly used storage drives are hard drives, floppy drives, optical drives, flash drives, and network drives. The capacity of a hard drive is dependent on many factors, including the number of disk sides, number of tracks per side, number of sectors on each track, and the amount of data that can be stored in each sector. “Drive interfaces” connect hard drives and optical drives to a computer. The connections of such drives require both a power cable and a data cable. The four most popular “input/output devices” used commonly with computers are the printer, monitor, mouse, and keyboard. The “bus” is a built-in electronic signal pathway in the motherboard to permit efficient and uninterrupted data transfer. A motherboard can have several buses, including the system bus, the PCI express bus, the PCI bus, the AGP bus, and the (outdated) ISA bus. “Ports” are the location at which external devices are connected to a computer motherboard. All commonly used peripheral devices, such as printers, scanners, and portable drives, need ports. A working knowledge of computers is necessary for the radiologist if the workflow is to realize its full potential and, besides, this knowledge will prepare the radiologist for the coming innovations in the ‘ever increasing’ digital future.

## Introduction

In the first half of this two-part article, we dwelt upon some fundamental concepts regarding computer hardware.[1] We had also briefly discussed the salient features of key hardware components in a computer, namely the motherboard, central processing unit (CPU), chipset, random access memory (RAM), and memory modules.

To recapitulate, a personal computer (PC) has a rectangular case that contains important components called hardware, many of which are integrated circuits (ICs). The fiberglass motherboard is the main printed circuit board and has a variety of hardware mounted on it, connected by electrical pathways called “buses.” The CPU is the largest IC on the motherboard; it contains millions of transistors that execute “programs.” The chipset controls data and the interaction of buses between the motherboard and the CPU. Memory modules (RAM) are semiconductor chips that store data and instructions for access by a CPU. RAM is classified by storage capacity, access speed, data rate, and configuration.

The second part of the article focuses on other important hardware items in a computer, namely storage drives, drive interfaces, input/output devices, buses, and ports.

## Storage Drives

Storage drive is a term describing a “memory” hardware used to store or retrieve data. The data may be text, images, audio, video, etc. A storage drive reads or writes data to storage media, which are classified as either spinning (CD/DVD) or linear media (tape) or, alternatively, as magnetic or optical. Storage memory is non-volatile and relatively slow.

**Figure 1:**
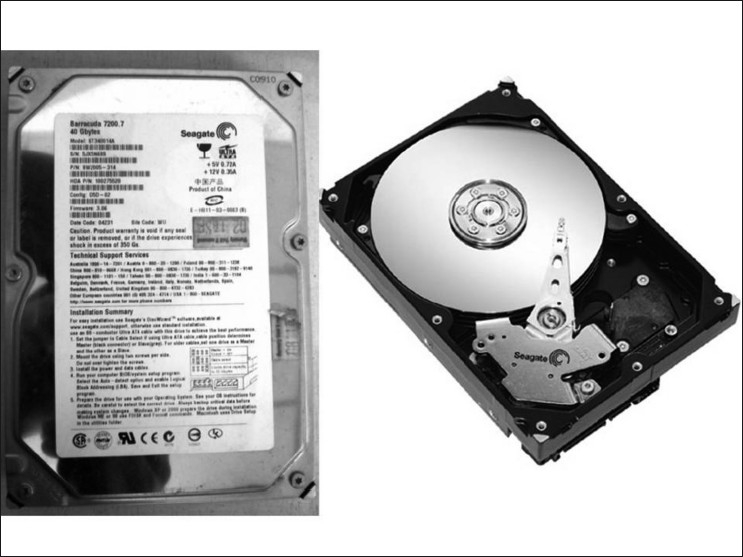
Main secondary storage device of a PC is a hard drive. This is a small case that contains magnetic-coated platters that rotate at high speed

Commonly used storage drives are hard drive, floppy drive, optical drive, flash drive, and network drive. A hard drive has a capacity [Figure 1], which is dependent on factors like a) number of disk sides, b) number of tracks per side, c) number of sectors on each track, and d) the amount of data that can be stored in each sector.[2,3]

The efficiency of a hard drive can be improved by increasing its speed and by allowing more data to be stored in it, which can be achieved by a) deleting unnecessary files and b) defragmenting the drive. Defragmentation, in simple words, is converting fragmented (non-contiguous) data into contiguous data clusters within a hard drive.[3]

Portable storage drives are versatile; they can be easily carried about and can be connected to any computer using USB, FireWire^®^, or SCSI ports.[4]

### Issues relevant to radiology practice

One should pay attention to a few important points with regard to storage when purchasing machines like USG, CT scan, MRI, and PET/CT scanners. The main computer system and dedicated workstations of modalities like CT scan and MRI should ideally have a large storage capacity. This is needed to partition and allot areas within the hard disk space for specialized functions, system software, images folders, offline working, backup data, etc. Furthermore, the availability of a large storage drive not only enables efficient handling of the assorted requirements mentioned above but also allows the machine to hold a large amount of images of patients, without the need to delete images periodically to prevent disk overload.

Another important aspect is regarding issues related to image storage for a given modality. The capacity of a storage drive is often expressed objectively in terms of the number of images of a 256 × 256 or 512 × 512 matrix that can be stored on the drive. This is useful when comparing hard disk capacities of different vendors during the purchase of sophisticated radiology equipment.[5]

## Drive Interfaces

Drive interfaces connect hard drives and optical drives to the computer. The connections of such drives require both a power cable and a data cable. Data cables eventually connect the drives to a drive controller, which is located on the motherboard. Some common interfaces are shown in Table 1, along with the acronyms used for them.

**Table 1:** Key features of drive interfaces (connects computers to hard disk drives)[6]

Type of drive interface	Abbreviation	Features
Integrated drive electronics (IDE)	ATA	Advanced technology attachment
Enhanced integrated drive electronics (EIDE)	ATA-2	Updated version; EIDE supports hard drives larger than 512 MB; enables direct memory access (DMA) for speed; uses attachment packet interface (ATAPI) for adding optical drives and tape drives
Parallel ATA	PATA	Parallel version of ATA drive controller interface
Serial ATA	SATA	Serial version of ATA drive controller interface
Small computer system interface	SCSI	Connect up to 15 drives (including internal and external)

### Issues relevant to radiology practice

Serial ATA (SATA) hard drives are preferred over parallel ATA (PATA) hard drives because of the following advantages: a) they provide more bandwidth; b) they are easy to install; c) they allow faster speeds; and d) they use smaller data and power cables. SATA drives are the most popular drives in the market today.[7]

SCSI is an intelligent, peripheral, peer-to-peer interface. It is popular on high-performance workstations and RAID (redundant array of independent disks) servers. Desktop computers and notebooks nowadays generally use ATA/IDE (advanced technology attachment standard interface / integrated drive electronics interface) or the newer SATA interfaces for hard disks, with USB and FireWire^®^ connections for external devices.

## Input/Output Devices

The functions of computers have increased gradually over time. From being used for simple functions like data and number processing in the early days, computers today perform a variety of functions: they handle audio and video formats, are vital to networks and connectivity using wired or wireless technologies, and they facilitate the addition of useful accessories like modems, tapes, drives, etc. These varied functions have increased the demand for different types of input/output hardware.

The four most popular input/output devices used commonly with computers are the printer, monitor, mouse, and keyboard.[8] Among input devices, other examples include microphone, camera, and touch screen. Important output devices are display monitors, printer, plotter, and audio speakers.

The extended and enhanced functions need hardware components termed “adapter cards,” which add controllers for these specific devices. In simple terms, adapter cards expand a computer’s capability. Typical examples are sound adapter cards, video adapter cards, network interface card (NIC), modem adapter cards, and SCSI adapter cards for connecting SCSI devices.

## Bus

**Figure 2:**
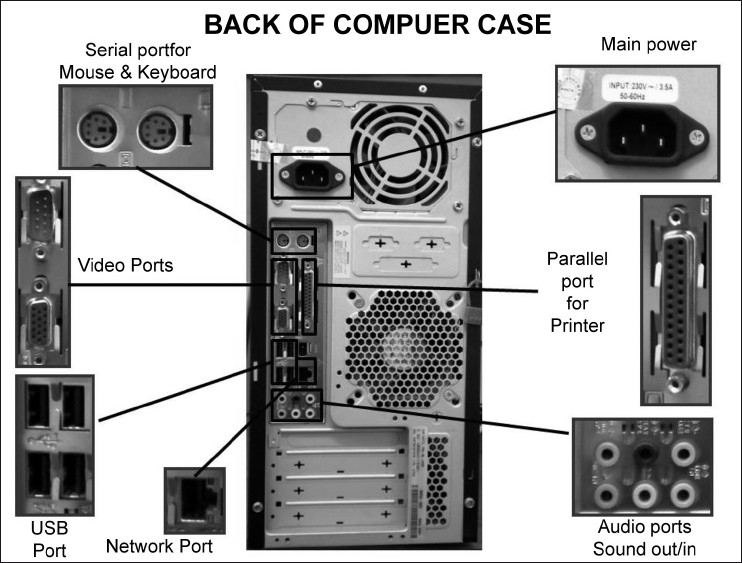
Back of a computer, showing important parts. The input and output devices are interfaced here

There is a need for an efficient, fast, and accurate method of moving data between the CPU and the many motherboard components. This is achieved practically by means of a bus. The bus is an electronic signal pathway that is built-in in the motherboard. It permits efficient and uninterrupted data transfer. They are also referred to as data channels or data pathways. A motherboard can have several buses, including the system bus, the PCI express bus, the PCI bus, the AGP bus, and the (outdated) ISA bus.[9] For adding external devices like monitor, telephone line, printer, etc., external buses are employed, which use expansion slots connected to ports at the back of the computer [Figure 2].[10] The different types of expansion slots are given in Table 2.

**Table 2:** Types of expansion slots

Types of expansion slots	Abbreviation	Features	Data transfer
Industry standard architecture	ISA	Older technology; seldom used	8-bit and 16-bit
Peripheral component interconnect	PCI	Standard slot currently used in computers	32-bit
PCI-express	PCI-E	Backward compatible with PCI slots	64-bit

## Ports

**Figure 3:**
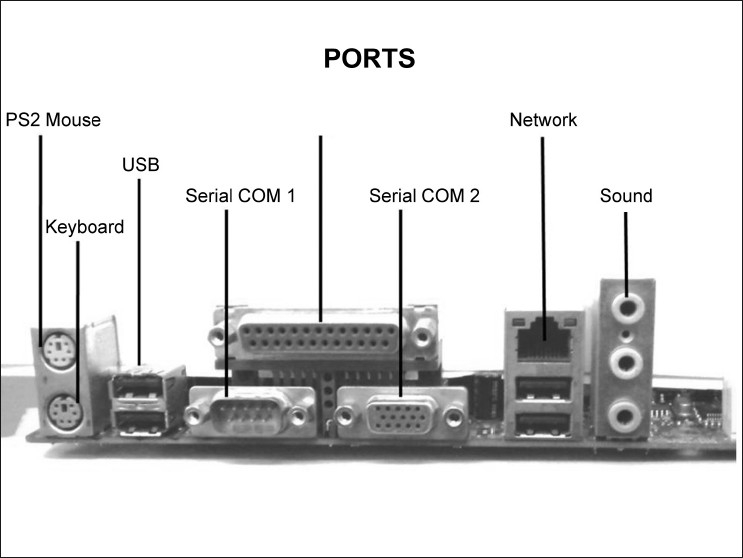
Ports. These are the locations at which external devices like printers, scanners, and portable drives are connected to a computer’s motherboard. Different types of ports are available, such as serial, parallel, and universal serial bus (USB). The USB is expected to replace many varieties of serial and parallel ports

Ports are the locations where external devices are connected to a computer motherboard. All commonly used peripheral devices like printers, scanners, and portable drives need ports. To facilitate the connection of assorted devices, different types of ports are available: serial, parallel, universal serial bus (USB), and SCSI ports [Figure 3]. Their key features are shown in Table 3. Other ports that are available include FireWire^®^, LAN (network), PS/2, and audio and video ports.[11]

**Table 3:** Commonly used ports

Type of ports	Speed transmission rates	Connectable devices
Serial	1 bit of data at a time	Modem, printer
Parallel	8 bits of data at a time	
Universal serial bus	USB 1.1 in full-speed mode: up to 12 Mbps; in low-speed mode: 1.5 Mbps	Computers, cameras, printers, scanners, storage devices, etc.
	USB 2.0 – up to 480 Mbps	
	USB 3.0 – 625 Mbps	
SCSI	Transmit data in excess of 320 Mbps	Support up to 15 devices

Parallel ports (seen in earlier machines) can be used to connect printers to computers but now these are connectable at the faster USB ports as well. Serial ports are used to connect low-speed peripherals like modems, mouse, and some types of scanners to the computer. Items that were once connected via parallel and serial ports are now increasingly being connected using USB ports.

The SCSI port supports a number of peripherals, e.g., hard disks, tape drives, CD-ROM drives, scanners, etc. FireWire^®^ is used to connect digital-video devices like camcorders or digital recorders to a computer. Other ports that are available include LAN (network), PS/2, and audio and video ports.

USB ports are used in general purpose interfacing of nearly 130 different types of external devices. They are increasingly becoming the preferred connecting points. External devices that can be connected using USB ports include mouse, keyboards, printers, media players, flash drives, and external hard drives, as well as expansion slots. Other important devices like digital cameras, smart phones, PDAs, and video game consoles can also be interfaced. The USB is expected to replace many varieties of serial and parallel ports. Some salient features of USB ports are listed in Table 4.

**Table 4:** Features of an USB port[12]

An external bus standard; USB 1.0 introduced in January 1996; USB 2.0 in April 2000; and USB 3.0 in 2010; Used in general purpose external high-speed device interfacing; Enhances plug-and-play of peripherals connected to a PC; Fully backward compatible; Very easy to install/uninstall; not attached by screws; USB are hot swappable, i.e., may be connected/disconnected even without shutting down the computer

Issues relevant to radiology practice

Transferring of digital movie and camera flash card data is expected to see a dramatic improvement with the introduction of USB 3.0. For instance, the transfer of a file size of 25 GB, which takes approximately 9 h with USB 1.0 and 14 min with USB 2.0, will take just 70 s with USB 3.0.[13] External devices using USB 3.0 are expected to reduce backup times considerably, improving productivity and efficiency.

Nevertheless, being easy to use and small in size, the USBs can be covertly used for transferring sensitive or confidential data and they may facilitate malware entry and easy transfer of viruses. Hence, USBs are banned by some vendors as well as administrators, for use on main consoles and workstations.

## Keyboard

This is an assembly of buttons, each representing the function, letter, or number engraved on it. Keyboards are useful devices for inputting text. English-language keyboards have a layout that is based on the QWERTY format seen in the early typewriters. For use with computers newer functions have been assigned to the individual buttons, with function-, number-, arrow-, or operating system specific keys. Further, pressing two or more keys in combination can assist specific functions.

### Mouse

It is a small, graphical user interface device with useful features. It is manually used for multiple tasks, such as placing text, clicking on buttons, dragging objects on screen, scrolling a page, and for controlling the pointer on the screen. The mouse may have two or three buttons, enabling a left-, middle-, or right-click function. It may have a scroll wheel that can be rotated using the fingers or be pressed down (thus allowing it to be used as a middle button). Newer mouse uses optical technology and need no rollers, directly tracing movement; they are much more accurate.[14]

## Recent Developments

The development of newer microprocessors is influencing the way PCs are marketed. Most of these are related to the original Intel Core which “is a brand name used for various mid-range to high-end consumer and business microprocessors.”[15] The core processors are more powerful than entry-level Celeron and Pentium processors.

In a nutshell, the latest computers marketed with Intel processors can be categorized into three groups.

The first is the popular lineup of core processors such as the Intel Core i7, Intel Core i5, and Intel Core i3.[15–17]

Likewise, the second category of the Intel^®^ Xeon^®^ processor 3000 sequence, 5000 sequence, and 7000 sequence[18] meets the needs of the server and workstation markets.

The third category of the Intel^®^ Atom™ processor enables a broad range of devices like tablets, netbooks, smartphones, handhelds, and entry-level desktop PCs.[19]

## Summary and Conclusion

A digital transformation has taken place in the practice of radiology and imaging due to the unprecedented impact of computers and their applications. As a result, computers are now an integral part of many activities within a radiology department. It has impacted the complete continuum of radiology workflow, including scheduling, image acquisition, image viewing, processing, analyzing and post-processing, computer-aided detection (CAD), multimedia integration, voice dictation, reporting, billing, transmitting, networking, electronic storage, picture archiving and communication systems (PACS), hospital information systems (HIS), radiology information systems (RIS), and teleradiology.

A working knowledge of computers will not only facilitate the use of the above technologies to their full potential in radiology workflow, but will also prepare radiologists to face an “ever-increasing” digital future.

## References

[1] Indrajit IK, Alam A (2010). Computer hardware for Radiologists: Part 2. Indian J Radiol Imaging.

[2] Hard Disk Drive.

[3] Computer Hard Drive. http://www.build-yourown-cheap-computer.com/computer-hard-drives.html.

[4] About FireWire IEEE1394. http://www.karbosguide.com/hardware/module5c3.htm.

[5] 3T MRI systems, Report 06006 Centre for Evidence-based Purchasing (CEP) NHS Purchasing and Supply Agency. http://www.pasa.nhs.uk/cep.

[6] Hard drive interface introduction and comparison. http://www.hdd-tool.com/hdd-basic/hard-drive-introduction-andcomparison.htm.

[7] SATA Hard Drive Buying Guide. http://www.suite101.com/content/sata-serial-ata-hard-drives-a28419.

[8] Input and Output Devices. http://www.freefree-computer-tips.info/computer-hardware/input-and-output-devices.html.

[9] The Bus. http://www.pccomputernotes.com/system_bus/bus01.htm.

[10] The Types of PC Expansion Slots. http://www.dummies.com/how-to/content/the-types-of-pc-expansion-slots.html.

[11] http://www.learning-aboutcomputers.com/computer_ports.php.

[12] USB Port. http://www.everythingusb.com/.

[13] SuperSpeed USB 3.0: More Details Emerge. http://www.pcworld.com/article/156494/superspeed_usb_30_more_details_emerge.html.

[14] Mouse. http://en.wikipedia.org/wiki/Mouse_(computing).

[15] Intel Core. http://en.wikipedia.org/wiki/Intel_Core.

[16] Intel Officially Launches 32nm Core i3, i5, i7. http://www.tomshardware.com/news/Core-i3-i5-i7-Intel,9390.html.

[17] Intel Core Microprocessors. http://en.wikipedia.org/wiki/Intel_Core.

[18] Processors. http://www.intel.com/products/processor/index.htm.

[19] Intel^®^ Atom™ Processor.

